# Supplementary material for: Resolving the full spectrum of human genome variation using Linked-Reads
Source: Genome Res. 2019 Apr;29(4):635–45. doi: 10.1101/gr.234443.118 (PMC6442396; doi:10.1101/gr.234443.118)
Supplement: Supplemental Material [file supp_gr.234443.118_Supplemental_longranger-2.2.zip › longranger-2.2.tar.bz2!/lr2.2-public/lib/stan/cmdstan/stan/doc/api/html/expression07__grammar_8hpp_source.html]

Stan: src/stan/gm/grammars/expression07\_grammar.hpp Source File


|  |  |
| --- | --- |
|  | Stan  2.5.0  probability, sampling & optimization |


- Main Page
- Related Pages
- Namespaces
- Classes
- Files

- File List
- File Members

All Classes Namespaces Files Functions Variables Typedefs Enumerations Enumerator Friends Macros Pages

- src
- stan
- gm
- grammars

expression07\_grammar.hpp

Go to the documentation of this file.

1 #ifndef STAN\_\_GM\_\_PARSER\_\_EXPRESSION\_GRAMMAR07\_\_HPP\_\_

2 #define STAN\_\_GM\_\_PARSER\_\_EXPRESSION\_GRAMMAR07\_\_HPP\_\_

3

4 #include <string>

5 #include <sstream>

6 #include <vector>

7

8 #include <boost/spirit/include/qi.hpp>

9

10 #include <stan/gm/ast.hpp>

11 #include <stan/gm/grammars/expression\_grammar.hpp>

12 #include <stan/gm/grammars/term\_grammar.hpp>

13 #include <stan/gm/grammars/whitespace\_grammar.hpp>

14

15 namespace stan {

16

17  namespace gm {

18

19  template <typename Iterator>

20  struct term\_grammar;

21

22  template <typename Iterator>

23  struct expression\_grammar;

24

25  template <typename Iterator>

26  struct expression07\_grammar

27  : public boost::spirit::qi::grammar<Iterator,

28  expression(var\_origin),

29  whitespace\_grammar<Iterator> > {

30

31  expression07\_grammar(variable\_map& var\_map,

32  std::stringstream& error\_msgs,

33  expression\_grammar<Iterator>& eg);

34

35  variable\_map& var\_map\_;

36

37  std::stringstream& error\_msgs\_;

38

39  term\_grammar<Iterator> term\_g;

40

41

42  boost::spirit::qi::rule<Iterator,

43  expression(var\_origin),

44  whitespace\_grammar<Iterator> >

45  expression07\_r;

46

47  };

48

49  }

50 }

51

52 #endif

expression\_grammar.hpp

stan::gm::expression\_grammar

**Definition:** expression07\_grammar.hpp:23

stan::gm::variable\_map

**Definition:** ast.hpp:439

stan::gm::expression07\_grammar::term\_g

term\_grammar< Iterator > term\_g

**Definition:** expression07\_grammar.hpp:39

stan::gm::expression07\_grammar::var\_map\_

variable\_map & var\_map\_

**Definition:** expression07\_grammar.hpp:35

stan::gm::expression07\_grammar::error\_msgs\_

std::stringstream & error\_msgs\_

**Definition:** expression07\_grammar.hpp:37

stan::gm::expression

**Definition:** ast.hpp:216

stan::gm::expression07\_grammar::expression07\_grammar

expression07\_grammar(variable\_map &var\_map, std::stringstream &error\_msgs, expression\_grammar< Iterator > &eg)

**Definition:** expression07\_grammar\_def.hpp:136

term\_grammar.hpp

ast.hpp

stan::gm::whitespace\_grammar

**Definition:** whitespace\_grammar.hpp:11

whitespace\_grammar.hpp

stan::gm::term\_grammar

**Definition:** expression07\_grammar.hpp:20

stan::gm::expression07\_grammar::expression07\_r

boost::spirit::qi::rule< Iterator, expression(var\_origin), whitespace\_grammar< Iterator > > expression07\_r

**Definition:** expression07\_grammar.hpp:45

stan::gm::expression07\_grammar

**Definition:** expression07\_grammar.hpp:26

stan::gm::var\_origin

int var\_origin

**Definition:** ast.hpp:411


---

[ Stan Home Page ]

*© 2011–2014,
Stan Development Team.*
